# New Metrics for Assessing the State Performance in Combating the COVID‐19 Pandemic

**DOI:** 10.1029/2021GH000450

**Published:** 2021-09-13

**Authors:** Yun Li, Megan Rice, Moming Li, Chengan Du, Xin Xin, Zifu Wang, Xun Shi, Chaowei Yang

**Affiliations:** ^1^ Department of Geography and GeoInformation Science George Mason University Fairfax VA USA; ^2^ NSF Spatiotemporal Innovation Center George Mason University Fairfax VA USA; ^3^ Department of Chemistry Carnegie Mellon University Pittsburgh PA USA; ^4^ Department of Epidemiology and Biostatistics University of California, San Francisco San Francisco CA USA; ^5^ School of Internal Medicine Yale University New Heaven CT USA; ^6^ Department of Geography Dartmouth College Hanover NH USA

**Keywords:** COVID‐19, hierarchical linear models, infection rate, performance evaluation, random effects

## Abstract

Previous research has noted that many factors greatly influence the spread of COVID‐19. Contrary to explicit factors that are measurable, such as population density, number of medical staff, and the daily test rate, many factors are not directly observable, for instance, culture differences and attitudes toward the disease, which may introduce unobserved heterogeneity. Most contemporary COVID‐19 related research has focused on modeling the relationship between explicitly measurable factors and the response variable of interest (such as the infection rate or the death rate). The infection rate is a commonly used metric for evaluating disease progression and a state's mitigation efforts. Because unobservable sources of heterogeneity cannot be measured directly, it is hard to incorporate them into the quantitative assessment and decision‐making process. In this study, we propose new metrics to study a state's performance by adjusting the measurable county‐level covariates and unobservable state‐level heterogeneity through random effects. A hierarchical linear model (HLM) is postulated, and we calculate two model‐based metrics—the standardized infection ratio (SDIR) and the adjusted infection rate (AIR). This analysis highlights certain time periods when the infection rate for a state was high while their SDIR was low and vice versa. We show that trends in these metrics can give insight into certain aspects of a state's performance. As each state continues to develop their individualized COVID‐19 mitigation strategy and ultimately works to improve their performance, the SDIR and AIR may help supplement the crude infection rate metric to provide a more thorough understanding of a state's performance.

## Key Points

- A hierarchical linear model was established to associate the infection rate with the collected explicit factors, which were demonstrated to greatly influence the spreading of COVID‐19 in previous studies, and the unobserved heterogeneity was also incorporated to better reflect the hierarchical structure
- Two model‐based metrics were proposed for assessing the state performance by adjusting the measurable county‐level covariates and the unobservable state‐specific variation
- These metrics can give insight into certain aspects of a state's performance in combating the COVID‐19 pandemic in addition to the widely used crude infection rate

## 1 Introduction

COVID‐19 first appeared in Wuhan, China at the end of 2019 (WHO, 2020). By early January, it had quickly spread to other countries and by March, the WHO claimed COVID‐19 a global pandemic. The first confirmed case in the United States (US) was reported in the state of Washington in late January (Kantis et al., 2020). By April, the US had become a global COVID‐19 epicenter (C. Yang et al., 2020) although COVID‐19 trends have varied among regions. For example, the state of New York was deemed an epicenter in April. By mid‐August, New York had managed to contain the spread of the disease; meanwhile, new hot spots appeared in California, Florida, and Texas (Adeline et al., 2020). In attempts to hinder the spread of the virus, the US governmental bodies at the federal, state, and local levels have implemented a series of containment and closure measures and public mandates including the closure of nonessential businesses and public spaces (Lee et al., 2020), as well as mask mandates and others (CDC, 2020).

Numerous contemporary studies have been conducted to examine the impacts of socioeconomic, medical, environmental, and policy factors on the transmission and mortality of COVID‐19. Hu et al. (2020) noted that racial segregation and overcrowding (i.e., more than one person in a room) increased the COVID‐19 incidence rate in Massachusetts. Mollalo et al. (2020) found that income variability and the proportion of black females were helpful in explaining the county‐level COVID‐19 incidence rates across the US. Wong and Li (2020) and Sun et al. (2020) investigated the impact of population density on the spread of COVID‐19. Liu et al. (2020) and Zhang et al. (2020) studied the association between meteorological factors (e.g., temperature) and the transmission of COVID‐19. Wu et al. (2020) found that high historical PM2.5 exposure was positively correlated with high COVID‐19 mortality rates. Liang et al. (2020) also discovered that urban air pollutants, especially NO_2_, led to more COVID‐19 cases. Others found that comorbidities attributed to chronic diseases (e.g., diabetes) and age had a considerable impact on the infection and fatality of COVID‐19 (Fu et al., 2020; Li et al., 2020; Priyadarsini & Suresh, 2020). Furthermore, containment and closure policies have been proven to be effective in reducing the growth rate of COVID‐19 (Courtemanche et al., 2020; Li et al., 2021; Matrajt & Leung, 2020).

In addition to explicit variables such as socioeconomic status, medical capacity, and policy factors, the spread of COVID‐19 is also related to unobservable heterogeneous sources which may impact the progression of the disease and the effectiveness of containment measures. The unobserved heterogeneity may be attributed to public knowledge, compliance, attitudes, personality, culture, etc., which are usually hard to directly measure or quantify. Besides, people residing in the same community/administrative division (e.g., state) who have similar backgrounds or other characteristics, such as gender, ethnicity, country of origin, or partisanship tend to be more similar. These similarities can be seen in people's consciousness of COVID‐19 and their willingness to comply with containment measures (Alahdal et al., 2020; Clements, 2020; Gollwitzer et al., 2020; Murphy & Moret‐Tatay, 2021; Priyadarsini & Suresh, 2020; Zhong et al., 2020).

The crude infection rate provides some indication of the performance of a state in regard to how well they are combating COVID‐19. Alternatively, in this study, an estimated infection ratio and the associated adjusted infection rate are used to learn the impact of unobserved sources of heterogeneity on the state‐level infection rate. The proposed measure is called the standardized infection ratio (SDIR), which adjusts the collected county‐level explicit factors and takes into account the state‐level unobserved heterogeneity. The SDIR reflects the state‐specific variation, which is unexplained by the explicit factors alone. In order to compare performances over time, the adjusted infection rate (AIR) was calculated by multiplying the SDIR by the national average infection rate among all the states. The crude and proposed infection rates may supplement each other in evaluating a state's performance as they evaluate effectiveness from different perspectives. However, the proposed metrics are advantageous in that they incorporate unobserved heterogeneous differences at the state‐level, ultimately giving more insight into the mitigation strategies for optimizing a state's performance.

## 2 Data

The outbreak of COVID‐19 in the US is the focus of this study. The case data used in this research included the cumulative daily number of county‐level confirmed cases from March to August in 2020, retrieved from the NSF Spatiotemporal Center COVID‐19 Data Repository (Sha et al., 2020), which is a compilation of data from sources such as USAFacts. We collected data of demographic and medical factors at both county and state levels (Table 1). The county‐level factors include the population density, and the proportions of White, African American, Hispanic/Latino, and seniors (age >60) in the population. The medical factors for which we have collected include the number of ICU beds, the proportion of people covered by insurance, the number of hospitals responding to COVID‐19, the proportions of the population with high blood pressure, diabetes, asthma, coronary heart disease, chronic kidney disease, and the proportion of the population who had received the influenza vaccine. When county‐level data was unavailable, state‐level information was used instead.

**Table 1:** Summary of the Explicit Factors Considered in This Study

Factor category	Variable name	Source	Description
Demographic	Population density	Harvard Dataverse	The spatial distribution of people in each county
	Proportion of the black race		The percent of people in the county who are black
	Proportion of the white race		The percent of people in the county who are white
	Proportion of the Hispanic population		The percent of people in the county who are Hispanic
	Proportion of older people		The percent of people in the county who are older than 60
Medical	ICU beds	Definitive Healthcare	The number of ICU beds at hospitals in the county
	Insurance coverage	United States Census	The proportion of people in the county whose medical care is covered by insurance
	Responding hospitals	Association of State and Territorial Health Officials (ASTHO)	The number of hospitals responding to COVID‐19 in the county
	Sufficient mask indicator		The difference between purchased masks and used masks by hospitals in the state
	Influenza vaccine	Center for Disease Control and Prevention	The proportion of people in the state with the influenza vaccine
	Asthma		The proportion of people in the state with asthma
	High blood pressure		The proportion of people in the state with high blood pressure
	Diabetes		The proportion of people in the state with diabetes
	Annual checkup		The proportion of people in the state who had a checkup in 2017
	Physical inactivity		The proportion of people in the state who participate in minimal physical activity
	Smokers		The proportion of people in the state who smoke
	Coronary heart disease		The proportion of people in the state with coronary heart disease
	Chronic kidney disease		The proportion of people in the state with chronic kidney disease
	COPD		The proportion of people in the state with COPD
Policy	Stringency Index	The University of Oxford	An aggregate score/composite measure indicating the overall policy strictness

Since the outbreak of the pandemic, governments worldwide have initiated a series of containment and closure policies in attempts to contain the rapid spread of COVID‐19. The University of Oxford has maintained a policy tracker to record the daily stringency of different types of policies for 184 countries since January 1, 2020. A stringency index (ranging from 0 to 100) was constructed by the data maintaining team by averaging several relevant individual measures, which indicates the strictness of the overall containment and closure measures. In addition to the demographic and medical factors summarized in Table 1, we included the daily stringency index recorded for the US (collected from the OxCGRT policy tracker repository) as an explicit factor to adjust; more details regarding the policy data can be found in Hale et al. (2020) and Hale et al. (2021).

We calculated the number of monthly cumulative confirmed cases by taking the total cumulative confirmed cases on the last day of each month and subtracting the total cumulative cases at the end of the previous month. We also calculated the crude infection rate by dividing the number of monthly cumulative confirmed cases in each county by the total population of that county.

## 3 Methods

### 3.1 Hierarchical Linear Models and State‐Specific Heterogeneity

In this study, we used hierarchical linear models (HLMs) (Raudenbush & Bryk, 2002) to estimate state‐specific effects from all county‐level data. HLMs are extensions of linear regression models which account for the clustered effect. Before proceeding to describe our model, we will first briefly review the HLM and related concepts in regression analysis. Multiple linear regression models are widely used to study the association between a set of independent variables (aka explanatory variables, predictors, covariates) and the dependent variable (aka response/outcome variable). For theoretical reasons, multiple linear regression models are usually intended for data with independent observations. With repeated measurements, there is likely to be a grouping effect within clusters formed by the observational units. The HLM provides an effective and parsimonious modeling strategy by incorporating random effects to account for the clustering/grouping effect (Garson, 2013; Guo, 2005; Nezlek & Zyzniewski, 1998; Sullivan et al., 1999; Woltman et al., 2012). Depending on the context, HLMs are also called mixed‐effects models in biometric applications (Fitzmaurice et al., 2011), multilevel models (Goldstein, 2011), and random coefficients models (Longford, 1993) in social science.

Random effects models have been used extensively spanning over many aspects in contemporary COVID‐19 research. Tsai et al. (2020) used a mixed‐effects model to study the reproducing number. Olsen et al. (2020) estimated the pandemic vulnerability in the early phase in India using a hierarchical model accounting for social/demographic/group differentials among impact factors. Chen et al. (2020) studied the association between COVID‐19 infection and PM concentration using a random coefficients model. Kim and Kwan (2021) applied random intercept growth models to analyze longitudinal mobility data in transport geography. Gadarian et al. (2021) explored the partisanship difference in relation to public health behaviors, attitudes, and policy opinions in the US using a random intercept logit model under a multilevel framework. Accounting for spatial autocorrelations, Konstantinoudis et al. (2021) investigated the effect of long‐term NO_2_ and PM2.5 exposure on COVID‐19 mortality in England through Bayesian hierarchical models.

In our study, each state is treated as a cluster of counties. A cluster's property is determined by both the characteristics of individual members within the cluster and the cluster‐level variation. Therefore, the HLM framework provides a good fit for our analysis with an intrinsic county‐level and state‐level hierarchical structure. Specifically, we target the response variable as the county‐level crude infection rate (represented in the log scale), which is the total number of new COVID‐19 confirmed cases in a month divided by the total population of each county. We account for the between‐state heterogeneity by postulating a state‐specific random effect $\alpha_{i}$, which is assumed to follow a normal distribution with mean μ and variance $\tau^{2}$. Equation 1 defines the HLM, where $Y_{ij}$ denotes the crude infection rate for the *j*th county in the *i*th state:

(1) $$\begin{array}{l} \log\left(Y_{ij}\right)=\alpha_{i}+\beta^{\prime }Z_{ij}+\epsilon_{ij},\\ \alpha_{i}=\mu +\omega_{i},\;\omega_{i}∼\;N\left(0,\;\tau^{2}\right),\\ i=1,\text{…}\;,I,\;j=1,\text{…}\;,n_{i}. \end{array}$$

Here, I denotes the total number of states in the US, $n_{i}$ denotes the number of counties in state i, $Z_{ij}={\left(Z_{ij1},\;Z_{ij2},\;\text{…}\;,\;Z_{ijp}\right)}^{\prime }$ denotes a set of p‐dimensional county‐specific covariates (Table 1) derived from the data, and $\epsilon_{ij}$ is the residual. The state‐specific intercept $\alpha_{i}$ is comprised of μ, which represents the national average infection over all states in the sample, and $\omega_{i}$, which is the random component variance, $\tau^{2}$, reflects the between‐state variation. The unobserved state‐level heterogeneity is captured by the cluster‐specific random intercept, which is a widely used approach in real practice (Bartels, 2008; Bell et al., 2019). Although DC could be treated as a second‐level political division like a county, it is not a sub‐division of any state. Therefore, we exclude DC from our data analysis to avoid further issues and complications.

### 3.2 COVID‐19 Standardized Infection Ratio (SDIR)

Based on the national average and state‐specific estimates, two metrics—the SDIR and AIR, were calculated and used in the subsequent analyses to measure a state's performance in combating the COVID‐19 pandemic. By adjusting both the explicit factors and unobserved heterogeneity, these two metrics serve as a supplement to the crude infection rate which is a measure commonly used to describe COVID‐19 trends.

In our study, the SDIR compares the performance of a target state to its counterfactual counterpart (a mirror state to itself) at the national level, because it is unlikely to have another state with exactly the same county‐level covariates. Similar methods have been developed in medical care profiling and related studies (Normand et al., 1997; X. Yang et al., 2014). Specifically, the SDIR for state i, as defined in Equation 2, is calculated by taking the ratio of the sum of the predicted infection rate in each county within state i (Equation 3) to that of the expected infection rate in each county within the same state (Equation 4):

(2) $${\text{SDIR}}_{i}=\frac{\sum_{j=1}^{n_{i}}{\hat{p}}_{ij}}{\sum_{j=1}^{n_{i}}{\hat{e}}_{ij}},$$

(3) $${\hat{p}}_{ij}=\exp\left({\hat{\alpha }}_{i}+{\hat{\beta }}^{\prime }Z_{ij}\right),$$

(4) $${\hat{e}}_{ij}=\text{exp}\left(\hat{\mu }+{\hat{\beta }}^{\prime }Z_{ij}\right),$$

where the parameter estimates in Equations 3 and 4, namely, $\hat{\mu }$, $\left\{{\hat{\alpha }}_{1},{\hat{\alpha }}_{2},\text{…},{\hat{\alpha }}_{I}\right\}$, $\hat{\beta }$, and ${\hat{\tau }}^{2}$, are obtained from the HLM defined in Equation 1. The predicted rates are calculated for all counties in a certain state using the state‐specific estimates (i.e., ${\hat{\alpha }}_{i}$ for state i), while the expected rates are based on the national average estimate (i.e., $\hat{\mu }$ for the whole nation).

Conceptually, a mirror state was created for every state to aid in the understanding of the unobserved state‐level heterogeneity. Each state consists of the aggregated effects from counties and state‐specific characteristics that are unobservable. The mirror state could be regarded as a national performer who has the same explicit factors as the corresponding state but includes the national average intercept (μ) instead of a state‐specific random intercept ($\alpha_{i}$). This average intercept represents the effect quantified by the national average. The national performer adjusts this average performance to a specific state by accounting for that state's unique explicit circumstances. This helps define how well a specific state is doing with respect to the average state performance. In other words, the numerator in Equation 2 represents the predicted infection rate of the original state, while the denominator represents the estimated infection rate of the corresponding mirror state. The SDIR compares the performance of the original state and the corresponding mirror state in combating the spread of COVID‐19. A lower SDIR suggests a lower‐than‐expected outcome ratio, and hence, a better performance; a higher SDIR indicates a worse performance. Specifically, if the SDIR >1, the state is concluded to perform worse than the mirror state, indicating that the state could perform better. On the other hand, if the SDIR <1, the state performs better than the mirror state, exhibiting a good performance in combating COVID‐19 in regard to its current circumstance.

### 3.3 Adjusted Infection Rate (AIR)

SDIR is a ratio specific to a state in a specific month, that is, the mirror state playing the national average performer with the same explicit factors paired with the target state. Therefore, SDIRs are not comparable in other months for a state because these values are calculated based on models built with factors collected for a particular month. For example, a SDIR of 1.7 for a state in April does not necessarily mean a worse performance than a SDIR of 1.5 for the same state in June. However, with the help of another metric, the AIR, it is possible to compare a state's performance as the pandemic progresses. Deriving a suitable metric that describes a state's effectiveness over time is critical as it gives more insights into how a state's performance changes as the severity of the pandemic changes. The course of the pandemic not only included changes in epidemiological factors, but it also included changes in states' mitigation strategies. For example, policies typically became less stringent over time and there may have been a greater stress placed on medical resources. The AIR was calculated from the SDIR multiplied by the corresponding national crude infection rate in a month, which is the total number of infected individuals in the month divided by all the people in the country. Specifically, we calculated the adjusted rate, ${\text{AIR}}_{i}$ for state i in Equation 5, where $\bar{\text{IR}}$ denotes the national mean infection rate, and ${\text{SDIR}}_{i}$ is defined in Equation 2.

(5) $${\text{AIR}}_{i}={\text{SDIR}}_{i}\times \bar{\text{IR}}=\frac{\sum_{j=1}^{n_{i}}{\hat{p}}_{ij}}{\sum_{j=1}^{n_{i}}{\hat{e}}_{ij}}\times \bar{\text{IR}}$$

For a state i, ${\text{SDIR}}_{i}$ is a unitless ratio, calculated by adjusting for the county‐level covariates within the target state collected for a specific month and the model‐induced random effects estimates. The denominator of the ratio in Equation 2 is constructed based on the counterfactual state at the national level (i.e., the national performer) sharing exactly the same county‐level covariates. Multiplied by the national average infection rate $\bar{\text{IR}}$ as in Equation 5, for the same state i, the between‐month ${\text{AIR}}_{i}$ becomes meaningful and comparable. In other words, the SDIR can be viewed as an amplifying (or shrinking) factor for the national average (crude) infection rate measured for a specific month; any comparison between months is made relative to the national average infection rate for the same state in the same month.

## 4 Results

### 4.1 Crude Infection Rate

**Figure 1:**
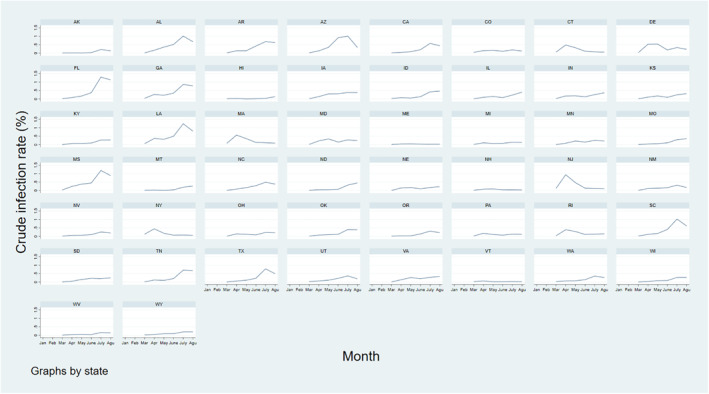
Crude infection rate time trend by state.

Trends in the state‐level crude infection rate varied widely throughout the study period. However, there were noticeable peaks in the infection rate at specific months (Figure 1). Most states saw a slight peak in the incidence rate in April and a maximum peak in July. In regard to state‐specific trends, northern states such as New Jersey, Massachusetts, and Delaware showed the highest infection rates in March and April. In June, Arizona had the highest infection rate which was particularly higher than any other state at this time. In the last 2 months of the study period, southern states such as Florida, Louisiana, and Mississippi had the highest infection rates. Analysis of the infection rate showed that the COVID‐19 epicenter shifted throughout the pandemic. Furthermore, the COVID‐19 infection rate peaked at different times depending on the region. Many northern states had a prominent peak in April which was typically the maximum infection rate these states reached during the analysis period. Similarly, the peak seen in many southern states in July often represented their maximum infection rate.

However, the crude infection rate describing a certain time period is only derived from the number of confirmed cases and the population of a state. Sometimes it is not fair to compare the crude infection rates of two states with different medical resources or social factors. For example, the state of New York may have better medical resources than the state of Connecticut. This emphasizes the importance of evaluating state performance from a supplemental perspective. In our study, we use the SDIR and AIR to account for the unobserved heterogeneity that may affect the infection rate.

### 4.2 SDIR in April and July and the AIR Trend

The SDIR adjusts the medical, social, and policy‐related factors as well as the state‐specific heterogeneity, which is typically neglected in crude infection rate calculations. It serves as an additional way to quantify the infection rate and helps in evaluating each state's performance in combating COVID‐19.

**Figure 2:**
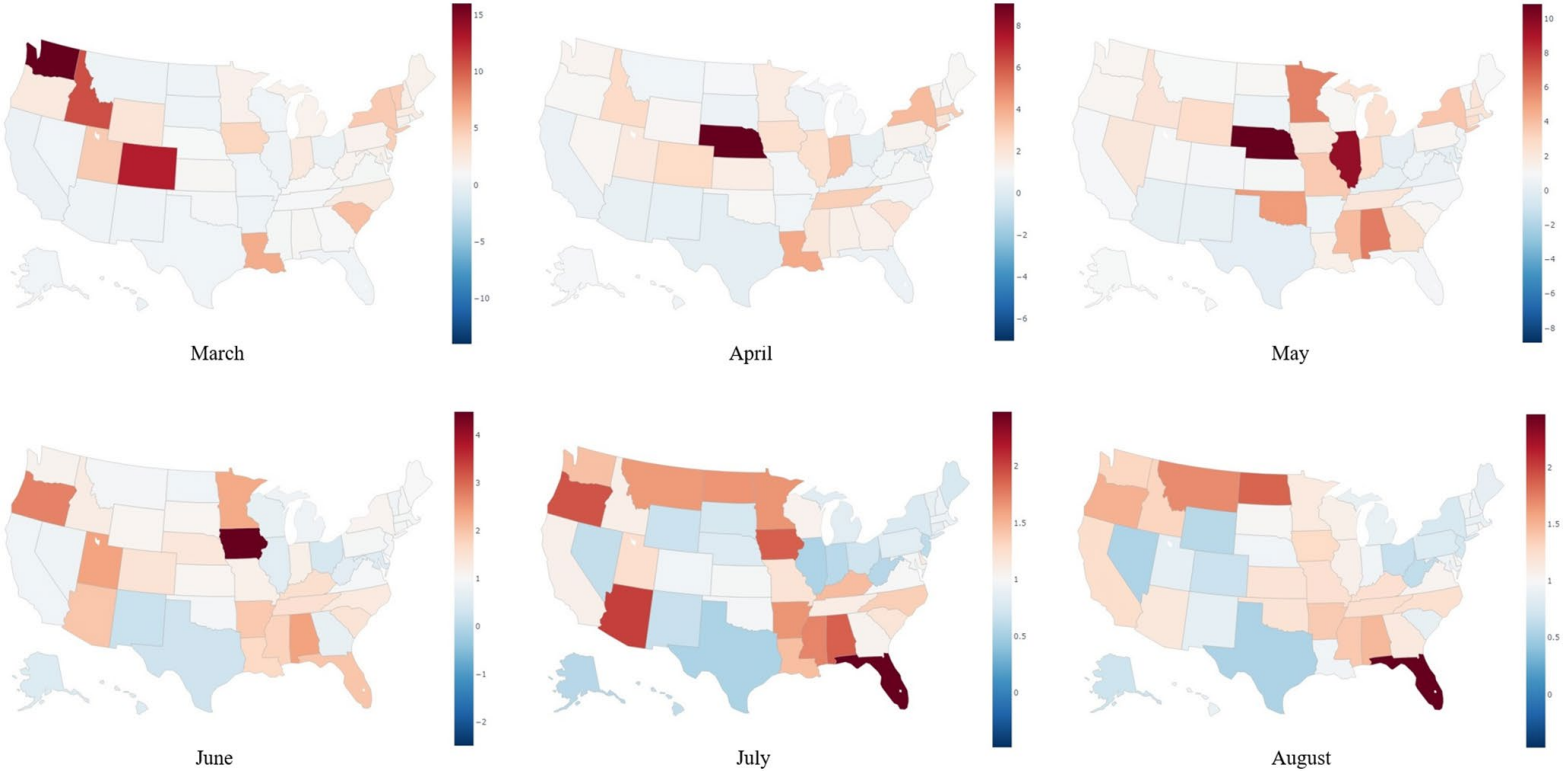
State standardized infection ratio values for every month in the analysis period.

The maps in Figure 2 show the SDIR values in all US states in each month of the study period. As explained previously, a SDIR value less than 1 (blue) means that the state performed well compared to its mirror state in a certain month. In contrast, a SDIR value greater than 1 (red) means that the state performed poorly compared to their expected ability. It is important to keep in mind that the SDIR models for each month are independent of each other and therefore cannot be compared over time. Therefore, the discussion of the SDIR results will focus on individual months in the analyses time frame.

**Figure 3:**
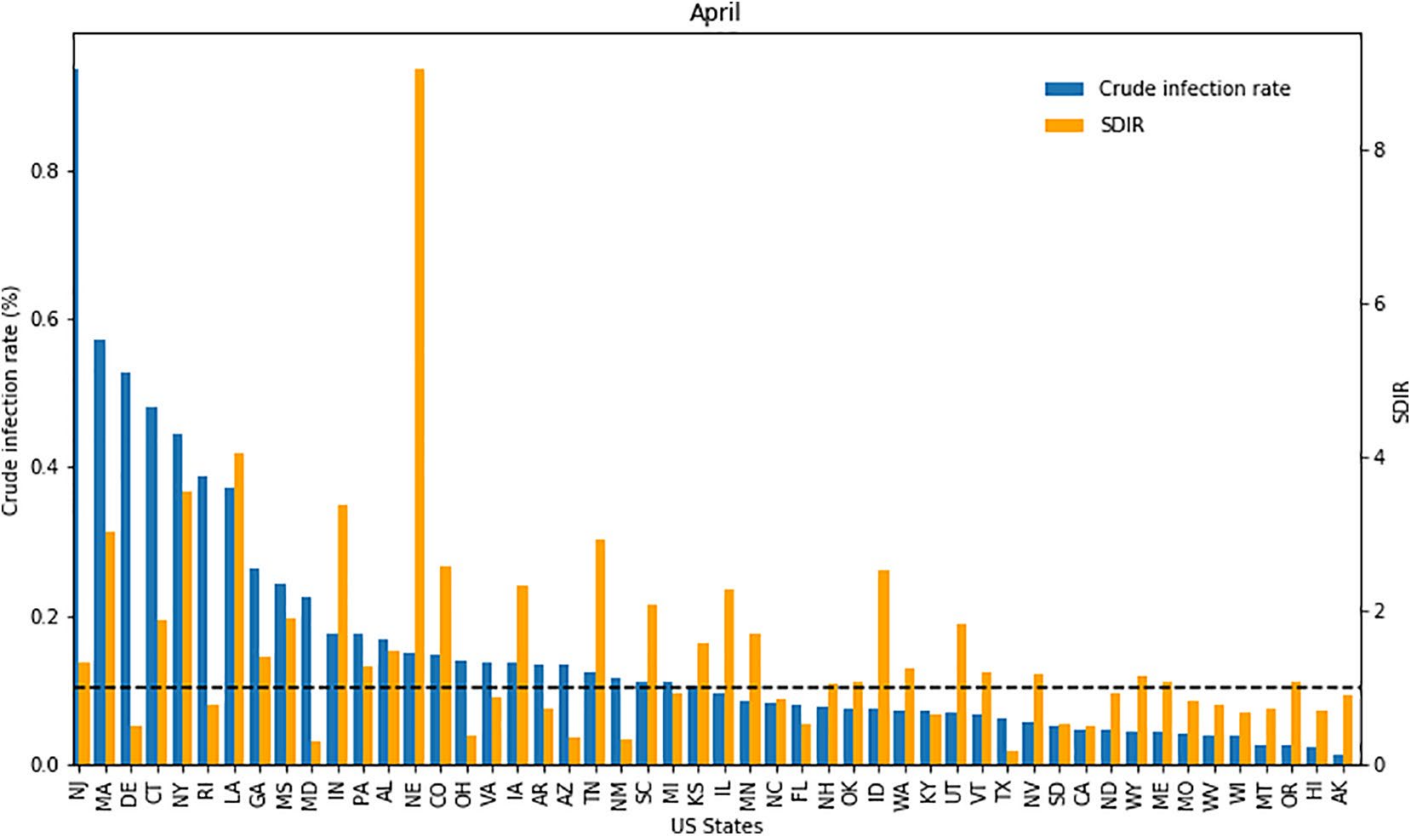
State standardized infection ratio values and infection rates in April.

Figure 3 shows that New Jersey, Massachusetts, Delaware, Connecticut, New York, and Rhode Island had some of the highest crude infection rates in April (0.94%, 0.57%, 0.53%, 0.48%, 0.44%, and 0.39%, respectively). However, the SDIR results are not always consistent with the crude infection rates. For example, New Jersey, Massachusetts, Connecticut, and New York had high (>1) SDIR values (1.34, 3.02, 1.87, and 3.56, respectively). Meanwhile, Delaware and Rhode Island had low (<1) SDIR values of 0.49 and 0.76 respectively. This trend is also seen in Figure 2 as Delaware and Rhode Island are blue while the other Northern states are red. Generally, one would assume that a high crude infection rate would indicate that the state performed poorly in combatting COVID‐19. However, the SDIR results show that a state's crude infection rate does not fully describe how effective it is in handling the pandemic.

**Figure 4:**
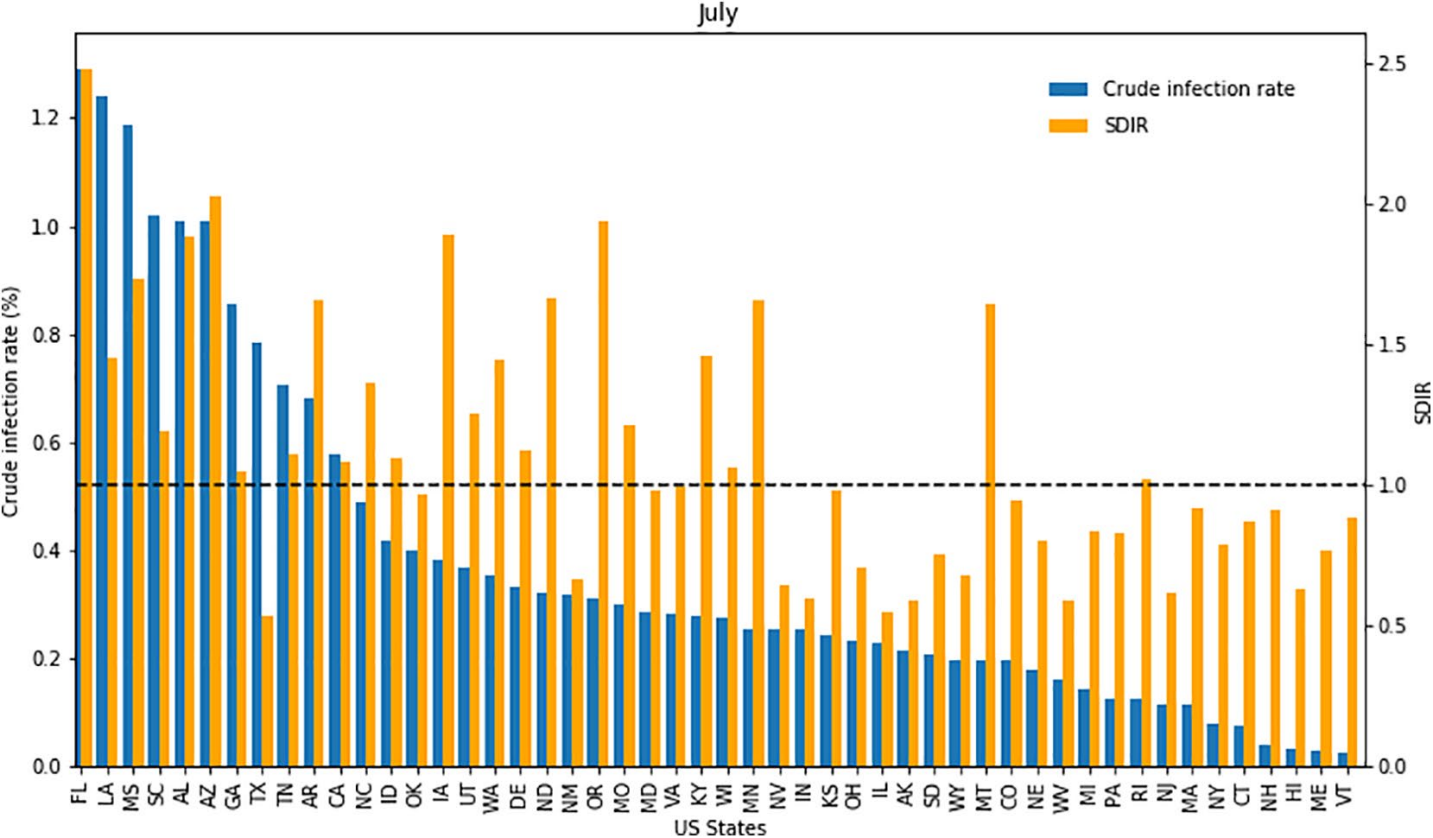
State standardized infection ratio values and infection rates in July.

More recent months also indicate that a high crude infection rate does not necessarily correspond to a high SDIR. For example, many southern states had their peak COVID‐19 crude infection rates in July (Figure 4). Some southern states like Florida and Louisiana had high infection rates (1.29% and 1.24%) and high SDIR values (2.48 and 1.45). However, Texas had a high infection rate (0.79%) but a low SDIR (0.54). Indeed, states in the same region often showed different levels of effectiveness as seen in the SDIR analysis for each individual month. Similar results can be seen in states that did not see infection rate spikes in July. For example, Rhode Island had a low infection rate (0.13%) but a high SDIR (1.02). Similarly, Georgia and Oklahoma did not see spikes in their infection rates in July, however, they showed higher infection rates (0.86% and 0.40%) than most other states. Despite these higher infection rates, the SDIR of Georgia (1.05) showed that it had a comparable performance in combatting COVID‐19 to the national average. The SDIR of Oklahoma was even lower (0.97). These low SDIR values suggest that these states performed better than one would expect by just looking at their crude infection rates. This may be the case if these states were able to effectively implement and enforce containment and closure policies, which is in turn dependent on other unobserved factors specific to a state such as the public awareness and willingness to comply. Indeed, Oklahoma set up extensive information campaigns, including public service announcements and social media graphics, in an attempt to educate the public about personal hygiene and COVID‐19 mitigation strategies (The University of Oklahoma, 2021). Public compliance and knowledge represent unobservable factors that may help states like Oklahoma and Georgia to perform well, in respect to the SDIR, despite having high infection rates.

**Figure 5:**
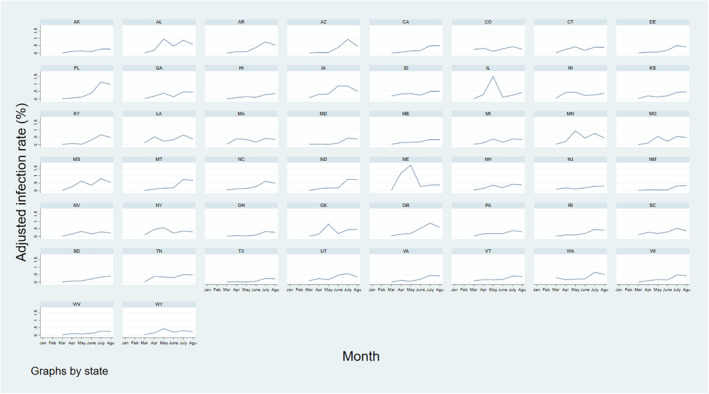
Adjusted infection rate trends by state from March to August in 2020.

The AIR metric was used to compare a state's performance as the pandemic progressed. The trends in the AIR throughout the analysis period revealed that some states had an increasingly worse performance over time. For example, Florida saw a noticeable rise in the AIR over most months of the analysis period (Figure 5) which means that it performed worse as the pandemic progressed. Florida's AIR values from March to August were 0.01%, 0.07%, 0.13%, 0.38%, 1.13%, and 0.97%. This is similar to Florida's infection rate trend which also steadily increased throughout most months in the analysis period. In this case, both metrics indicate that Florida was not performing well in combatting the virus. Although no states showed a steady improvement in performance over the months, which would be seen with constantly decreasing AIR values, many states showed a fluctuating AIR trend. For example, New York had AIR values of 0.09%, 0.46%, 0.58%, 0.21%, 0.36%, and 0.30% throughout consecutive months. On the other hand, New York's infection rate peaked in April and then decreased for the rest of the study period. Similarly, many southern states saw increasing infection rates that peaked in July (Figure 1), however, their AIR trends fluctuated throughout the analysis period. For example, Mississippi and Alabama saw a dip in their AIR in June even though their infection rates were still increasing. Decreasing AIR trends during these periods suggests that these states were combatting the virus more successfully than would be assumed by just considering their increasing infection rates. Furthermore, as the AIR fluctuated throughout the analysis period for most states, there were likely months when each state was not performing well in terms of this metric. For example, New York's infection rate started to steadily decrease after April, however, the AIR still increased from June to July.

## 5 Discussion

The crude infection rate is a typical measure of COVID‐19 widely used in practice. Although Connecticut and New York had similar infection rate time trends throughout the analysis period, this study finds that their SDIR performances were quite different. This shows that using multiple metrics that account for different types of information can be helpful in obtaining a more holistic picture of a state's performance. As the SDIR is able to account for the between‐state variation in addition to the county‐level explicit factors, utilizing the SDIR values alongside the crude infection rate could help provide additional information in estimating the effectiveness of each state as they work to combat COVID‐19. For example, Georgia and Oklahoma had high crude infection rates but relatively low SDIR in July. From only considering the crude infection rate, it may be assumed that more infections cause states like Georgia and Oklahoma to perform poorly. Through our model, Georgia and Oklahoma's estimated state‐level SDIR in July are 1.05 and 0.97, respectively, indicating that they performed comparable to the national average in July (in fact, the latter one is slightly better). By doing this, we can tell if a state is doing its best, or better than the national average, in combatting COVID‐19 instead of simply using the increased infections. When the crude infection rate and SDIR are used together, it is possible to see a state with a high infection rate that is, still effectively combating COVID‐19.

The SDIR and AIR values have implications in areas including policymaking. More mindful decisions can be made by using the SDIR and AIR metrics, alongside the infection rate, as they help estimate how effective a state is in combating COVID‐19. Additionally, these measures give insight into variation between states, which may affect COVID‐19 trends. Consequently, policies can focus on improving deficiencies in these areas. For example, state governments could focus on public information campaigns which could help make the population more knowledgeable about COVID‐19 and mitigation measures or create incentives which could make the public more willing to comply with policy mandates. Additionally, states could model their future mitigation strategies after months when they performed well, for example, their SDIR <1, by optimizing their resources and prioritizing their needs in the same way. Comparing changes in the AIR trends over time with infection rate spikes also reflects how easing restrictions and other factors (e.g., public altitude) affect a state's effectiveness. Governments need to be aware and mindful of the impacts of these events in developing their future mitigation strategies.

This study developed a hierarchical linear model and two model‐induced metrics (SDIR and AIR) which demonstrates the relationship between the infection rate and demographic, medical, and policy factors while simultaneously accounting for the unobserved state‐level heterogeneity. There are several limitations that can be improved in future research. First, similar to most existing research summarized in Section 3, spatial correlations were not considered in this study. As within cluster units may also exhibit dependence (i.e., counties within the same state tend to be more spatially correlated), accounting for spatial correlation might allow for a greater flexibility and generalizability of findings in the spatial context. Second, because the pandemic has experienced several waves (Kim & Kwan, 2021; H. Wang et al., 2021), evaluating the state performance by incorporating the wave effects can be much more informative, as some state may perform less promising in the beginning but improves as time progresses. Last but not least, other dynamic time‐varying factors such as the vaccination rates (Conlon et al., 2021; Levine‐Tiefenbrun et al., 2021), meteorological variables (J. Wang et al., 2021) and pollutant factors (Liang et al., 2020; López‐Feldman et al., 2021; Ogen, 2020; Yao et al., 2020) can also be assembled into the model establishment.

## 6 Conclusion

Numerous contemporary researches have been conducted to evaluate how different factors influence COVID‐19 trends in the US. Effects from explicitly measurable and collectable factors can be used to model and quantify their impacts on the crude infection rate as these variables may give some states advantages that make their COVID‐19 mitigation easier. However, it is hard to account for unobservable sources of factors that may introduce heterogeneous effects. Our study proposed additional metrics—the SDIR and AIR, which adjust the county‐level explicit factors and account for state‐level variation. This was achieved through building a hierarchical linear model with random effects. The SDIR measured how well a state was performing compared to its counterfactual national average performer under current circumstances at a specific month. The AIR was able to show a time trend of each state's effectiveness by taking into account the national crude infection rate throughout the pandemic.

Our analysis was significant as it showed that in some cases, states with a low infection rate in a specific month had a high SDIR. This can give insight into how a state could try to improve their performance. Similarly, the AIR showed that most states' performances fluctuated throughout the course of the pandemic. Compliance to mitigation measures often shifted throughout the pandemic as people became restless (Temple, 2020). At the same time, information regarding the virus has increased rapidly over time and may help increase the public's awareness. These new metrics can provide valuable insight into differentiating and evaluating the performance between states, and can be more informative when supplemented to the crude infection rate.

## Conflict of Interest

The authors declare no conflict of interest relevant to this study.

## Supporting information

Supporting Information S1

## Data Availability

The data used for building the HLM model in the study are available via https://doi.org/10.5281/zenodo.5081617.
