# Supplementary material for: New Metrics for Assessing the State Performance in Combating the COVID‐19 Pandemic
Source: Geohealth. 2021 Sep 13;5(9):e2021GH000450. doi: 10.1029/2021GH000450 (PMC8437244; doi:10.1029/2021GH000450)
Supplement: Supplementary file 1 — Supporting Information S1 [file GH2-5-e2021GH000450-s001.docx]

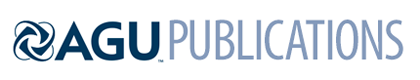


*Geohealth*

Supporting Information for

**New metrics for assessing the state performance in combating the COVID-19 pandemic**

Yun Li^1,2^, Megan Rice^3^, Moming Li^4^, Chengan Du^5^, Xin Xin^5^, Zifu Wang^1,2^, Xun Shi^6^, Chaowei Yang^1,2*^

^1^Department of Geography and GeoInformation Science, George Mason University, Fairfax, VA, USA

^2^NSF Spatiotemporal Innovation Center, George Mason University, Fairfax, VA, USA

^3^Department of Chemistry, Carnegie Mellon University, Pittsburgh, PA, USA

^4^Department of Epidemiology and Biostatistics, University of California at San Francisco, San Francisco, CA, USA

^5^School of Internal Medicine, Yale University

^6^Department of Geography, Dartmouth College

**Contents of this file**

Tables S1 to S5

**Introduction**

Tables in this supporting file show the parameter estimates from the HLMs for every month from March to August in 2020 as well as the crude infection rates, SDIRs, and AIRs calculated for every state in the study period.

Table S1. Parameter estimates from the HLMs (March to May in 2020).

|  | **March** | | **April** | | **May** | |
| --- | --- | --- | --- | --- | --- | --- |
| **Variables** | **Coefficient** | **p-value** | **Coefficient** | **p-value** | **Coefficient** | **p-value** |
| Population density | 0.000 | <.0001 | 0.000 | <.0001 | -0.001 | 0.005 |
| Proportion of the black race | -0.021 | <.0001 | 0.003 | 0.451 | 0.031 | <.0001 |
| Proportion of the white race | -0.036 | <.0001 | -0.018 | <.0001 | 0.009 | 0.042 |
| Proportion of the Hispanic population | 0.005 | 0.001 | 0.025 | <.0001 | 0.053 | <.0001 |
| Proportion of older people | -0.019 | 0.132 | -0.028 | 0.007 | -0.009 | 0.320 |
| ICU beds | 0.003 | <.0001 | 0.001 | <.0001 | 0.000 | 0.976 |
| Insurance coverage | 0.105 | 0.344 | 0.109 | 0.090 | 0.294 | 0.003 |
| Responding hospitals | -0.007 | 0.726 | 0.001 | 0.935 | -0.005 | 0.799 |
| Sufficient mask indicator | -0.537 | 0.482 | -0.512 | 0.307 | -1.533 | 0.040 |
| Influenza vaccine | -0.079 | 0.301 | 0.030 | 0.553 | 0.034 | 0.635 |
| Asthma | 0.074 | 0.783 | 0.114 | 0.574 | -0.192 | 0.488 |
| High blood pressure | -0.038 | 0.860 | -0.019 | 0.898 | 0.141 | 0.493 |
| Diabetes | -0.148 | 0.639 | -0.200 | 0.361 | -0.073 | 0.802 |
| Annual checkup | 0.038 | 0.655 | 0.086 | 0.112 | 0.161 | 0.033 |
| Physical inactivity | 0.201 | 0.118 | 0.196 | 0.020 | 0.265 | 0.034 |
| Smokers | 0.025 | 0.886 | 0.035 | 0.813 | 0.299 | 0.130 |
| Coronary heart disease | 0.138 | 0.891 | 0.140 | 0.826 | -0.621 | 0.483 |
| Chronic kidney disease | 0.390 | 0.809 | 0.059 | 0.955 | -2.572 | 0.078 |
| COPD | -0.182 | 0.735 | -0.230 | 0.537 | -0.155 | 0.768 |
| Stringency index | -0.050 | 0.140 | -0.006 | 0.687 | 0.001 | 0.953 |
| Intercept | -3.472 | 0.001 | -2.004 | 0.042 | -1.937 | 0.081 |

Table S2. Parameter estimates from the HLMs (June to August in 2020).

|  | **June** | | **July** | | **August** | |
| --- | --- | --- | --- | --- | --- | --- |
| **Variables** | **Coefficient** | **p-value** | **Coefficient** | **p-value** | **Coefficient** | **p-value** |
| Population density | -0.001 | 0.001 | 0.000 | 0.003 | -0.001 | 0.002 |
| Proportion of the black race | -0.005 | 0.035 | -0.001 | 0.413 | 0.011 | 0.000 |
| Proportion of the white race | -0.024 | <.0001 | -0.010 | <.0001 | 0.001 | 0.625 |
| Proportion of the Hispanic population | 0.017 | <.0001 | 0.007 | <.0001 | 0.004 | <.0001 |
| Proportion of older people | -0.020 | 0.007 | -0.017 | <.0001 | -0.038 | <.0001 |
| ICU beds | 0.000 | 0.448 | 0.000 | 0.057 | -0.001 | 0.019 |
| Insurance coverage | 0.031 | 0.502 | -0.035 | 0.263 | -0.016 | 0.558 |
| Responding hospitals | -0.004 | 0.709 | 0.001 | 0.856 | 0.001 | 0.893 |
| Sufficient mask indicator | 0.109 | 0.797 | 0.343 | 0.248 | 0.364 | 0.174 |
| Influenza vaccine | -0.034 | 0.361 | -0.038 | 0.136 | -0.030 | 0.178 |
| Asthma | -0.182 | 0.199 | -0.204 | 0.027 | -0.304 | 0.001 |
| High blood pressure | -0.027 | 0.796 | 0.034 | 0.623 | 0.048 | 0.470 |
| Diabetes | 0.103 | 0.496 | 0.152 | 0.132 | 0.076 | 0.411 |
| Annual checkup | 0.055 | 0.184 | 0.002 | 0.938 | 0.011 | 0.687 |
| Physical inactivity | -0.010 | 0.881 | -0.041 | 0.322 | -0.020 | 0.590 |
| Smokers | -0.087 | 0.356 | -0.056 | 0.358 | -0.023 | 0.686 |
| Coronary heart disease | -0.308 | 0.484 | -0.056 | 0.850 | -0.056 | 0.831 |
| Chronic kidney disease | 0.290 | 0.683 | -0.290 | 0.532 | -0.589 | 0.188 |
| COPD | 0.340 | 0.200 | 0.217 | 0.220 | 0.188 | 0.235 |
| Stringency index | -0.015 | 0.143 | -0.007 | 0.266 | -0.009 | 0.260 |
| Intercept | -1.500 | 0.017 | -1.014 | 0.015 | -1.196 | 0.002 |

Table S3. Crude infection rate by state from March to August in 2020.

| **State** | **March** | **April** | **May** | **June** | **July** | **August** |
| --- | --- | --- | --- | --- | --- | --- |
| AK | 0.006 | 0.014 | 0.007 | 0.029 | 0.214 | 0.137 |
| AL | 0.015 | 0.169 | 0.357 | 0.513 | 1.011 | 0.676 |
| AR | 0.018 | 0.135 | 0.138 | 0.419 | 0.683 | 0.633 |
| AZ | 0.017 | 0.134 | 0.344 | 0.910 | 1.010 | 0.331 |
| CA | 0.016 | 0.047 | 0.084 | 0.203 | 0.577 | 0.434 |
| CO | 0.045 | 0.147 | 0.168 | 0.108 | 0.195 | 0.120 |
| CT | 0.058 | 0.481 | 0.321 | 0.111 | 0.075 | 0.059 |
| DE | 0.031 | 0.528 | 0.535 | 0.186 | 0.332 | 0.222 |
| FL | 0.015 | 0.081 | 0.170 | 0.368 | 1.292 | 1.127 |
| GA | 0.035 | 0.263 | 0.215 | 0.342 | 0.857 | 0.774 |
| HI | 0.011 | 0.024 | 0.002 | 0.011 | 0.034 | 0.132 |
| IA | 0.011 | 0.137 | 0.298 | 0.305 | 0.380 | 0.373 |
| ID | 0.029 | 0.074 | 0.053 | 0.131 | 0.419 | 0.463 |
| IL | 0.007 | 0.097 | 0.144 | 0.076 | 0.228 | 0.400 |
| IN | 0.020 | 0.176 | 0.184 | 0.125 | 0.253 | 0.363 |
| KS | 0.008 | 0.106 | 0.177 | 0.094 | 0.243 | 0.314 |
| KY | 0.006 | 0.071 | 0.077 | 0.095 | 0.277 | 0.282 |
| LA | 0.061 | 0.372 | 0.318 | 0.502 | 1.242 | 0.798 |
| MA | 0.082 | 0.572 | 0.361 | 0.134 | 0.115 | 0.089 |
| MD | 0.017 | 0.225 | 0.343 | 0.157 | 0.285 | 0.239 |
| ME | 0.011 | 0.044 | 0.046 | 0.036 | 0.028 | 0.028 |
| MI | 0.017 | 0.110 | 0.071 | 0.081 | 0.142 | 0.136 |
| MN | 0.010 | 0.085 | 0.221 | 0.159 | 0.254 | 0.217 |
| MO | 0.009 | 0.042 | 0.061 | 0.108 | 0.298 | 0.355 |
| MS | 0.033 | 0.242 | 0.375 | 0.443 | 1.187 | 0.881 |
| MT | 0.012 | 0.025 | 0.005 | 0.037 | 0.196 | 0.259 |
| NC | 0.010 | 0.083 | 0.169 | 0.285 | 0.487 | 0.380 |
| ND | 0.012 | 0.046 | 0.050 | 0.072 | 0.321 | 0.451 |
| NE | 0.004 | 0.150 | 0.179 | 0.095 | 0.180 | 0.230 |
| NH | 0.016 | 0.078 | 0.092 | 0.043 | 0.040 | 0.031 |
| NJ | 0.124 | 0.937 | 0.468 | 0.138 | 0.115 | 0.104 |
| NM | 0.008 | 0.117 | 0.136 | 0.166 | 0.316 | 0.178 |
| NV | 0.009 | 0.056 | 0.063 | 0.097 | 0.253 | 0.198 |
| NY | 0.125 | 0.444 | 0.177 | 0.065 | 0.077 | 0.057 |
| OH | 0.010 | 0.140 | 0.117 | 0.084 | 0.230 | 0.212 |
| OK | 0.010 | 0.076 | 0.095 | 0.117 | 0.401 | 0.385 |
| OR | 0.009 | 0.025 | 0.029 | 0.138 | 0.310 | 0.211 |
| PA | 0.020 | 0.176 | 0.106 | 0.070 | 0.126 | 0.115 |
| RI | 0.032 | 0.388 | 0.285 | 0.110 | 0.125 | 0.144 |
| SC | 0.018 | 0.112 | 0.169 | 0.404 | 1.020 | 0.611 |
| SD | 0.009 | 0.052 | 0.155 | 0.221 | 0.206 | 0.251 |
| TN | 0.012 | 0.123 | 0.097 | 0.209 | 0.706 | 0.673 |
| TX | 0.005 | 0.063 | 0.109 | 0.224 | 0.785 | 0.494 |
| UT | 0.027 | 0.070 | 0.111 | 0.221 | 0.368 | 0.188 |
| VA | 0.009 | 0.138 | 0.268 | 0.200 | 0.282 | 0.332 |
| VT | 0.030 | 0.068 | 0.020 | 0.021 | 0.026 | 0.020 |
| WA | 0.033 | 0.072 | 0.075 | 0.141 | 0.355 | 0.271 |
| WI | 0.009 | 0.038 | 0.087 | 0.093 | 0.275 | 0.279 |
| WV | 0.006 | 0.040 | 0.048 | 0.038 | 0.160 | 0.142 |
| WY | 0.018 | 0.044 | 0.089 | 0.093 | 0.196 | 0.195 |

**Table S4.** SDIR by state from March to August in 2020.

| **State** | **March** | **April** | **May** | **June** | **July** | **August** |
| --- | --- | --- | --- | --- | --- | --- |
| AK | 0.565 | 0.888 | 0.955 | 0.521 | 0.586 | 0.693 |
| AL | 1.201 | 1.486 | 6.078 | 2.420 | 1.882 | 1.480 |
| AR | 0.279 | 0.737 | 0.552 | 1.933 | 1.656 | 1.380 |
| AZ | 0.376 | 0.357 | 0.273 | 1.979 | 2.024 | 1.160 |
| CA | 0.036 | 0.508 | 0.916 | 0.897 | 1.082 | 1.256 |
| CO | 12.794 | 2.564 | 0.775 | 1.494 | 0.945 | 0.682 |
| CT | 1.536 | 1.870 | 2.668 | 0.944 | 0.872 | 0.961 |
| DE | 0.981 | 0.493 | 0.480 | 0.985 | 1.125 | 1.031 |
| FL | 0.489 | 0.537 | 0.807 | 1.979 | 2.480 | 2.468 |
| GA | 1.024 | 1.410 | 2.421 | 0.732 | 1.050 | 1.146 |
| HI | 0.209 | 0.702 | 0.953 | 0.542 | 0.631 | 0.911 |
| IA | 4.246 | 2.317 | 2.132 | 4.493 | 1.886 | 1.286 |
| ID | 10.729 | 2.514 | 2.333 | 1.269 | 1.093 | 1.322 |
| IL | 0.373 | 2.267 | 9.631 | 0.592 | 0.551 | 1.082 |
| IN | 2.503 | 3.369 | 2.868 | 1.199 | 0.597 | 0.954 |
| KS | 1.225 | 1.586 | 0.854 | 1.080 | 0.978 | 1.208 |
| KY | 0.980 | 0.652 | 0.201 | 1.575 | 1.460 | 1.229 |
| LA | 6.519 | 4.046 | 1.523 | 1.695 | 1.449 | 0.946 |
| MA | 2.267 | 3.024 | 2.244 | 0.945 | 0.914 | 0.896 |
| MD | 1.742 | 0.309 | 0.115 | 0.559 | 0.978 | 0.936 |
| ME | 1.729 | 1.064 | 1.010 | 1.003 | 0.765 | 0.877 |
| MI | 1.688 | 0.930 | 2.459 | 0.859 | 0.835 | 0.895 |
| MN | 1.758 | 1.687 | 5.909 | 2.337 | 1.658 | 1.141 |
| MO | 0.430 | 0.834 | 3.576 | 1.186 | 1.210 | 1.228 |
| MS | 0.769 | 1.899 | 4.086 | 1.804 | 1.729 | 1.389 |
| MT | 0.300 | 0.729 | 0.968 | 0.938 | 1.641 | 1.697 |
| NC | 2.297 | 0.847 | 0.802 | 1.328 | 1.359 | 1.234 |
| ND | 0.363 | 0.920 | 1.078 | 0.893 | 1.662 | 1.861 |
| NE | 0.920 | 9.056 | 10.846 | 1.408 | 0.802 | 0.955 |
| NH | 1.860 | 1.049 | 2.290 | 0.979 | 0.912 | 0.934 |
| NJ | 4.636 | 1.337 | 0.619 | 0.920 | 0.618 | 0.747 |
| NM | 0.329 | 0.329 | 0.246 | 0.220 | 0.667 | 0.869 |
| NV | 0.464 | 1.180 | 2.095 | 0.847 | 0.643 | 0.555 |
| NY | 4.893 | 3.559 | 3.724 | 1.106 | 0.785 | 0.766 |
| OH | 0.248 | 0.374 | 0.174 | 0.425 | 0.701 | 0.669 |
| OK | 0.729 | 1.081 | 5.203 | 0.932 | 0.966 | 1.200 |
| OR | 2.497 | 1.072 | 1.215 | 2.749 | 1.935 | 1.521 |
| PA | 1.600 | 1.271 | 1.215 | 0.945 | 0.829 | 0.801 |
| RI | 0.644 | 0.763 | 0.583 | 0.990 | 1.016 | 1.020 |
| SC | 5.480 | 2.078 | 1.244 | 1.476 | 1.192 | 0.886 |
| SD | 0.281 | 0.529 | 0.524 | 1.085 | 0.751 | 1.016 |
| TN | 0.807 | 2.935 | 2.164 | 1.546 | 1.106 | 1.243 |
| TX | 0.472 | 0.185 | 0.024 | 0.288 | 0.537 | 0.545 |
| UT | 4.873 | 1.819 | 1.052 | 2.407 | 1.250 | 0.862 |
| VA | 0.769 | 0.875 | 0.371 | 0.960 | 0.991 | 1.044 |
| VT | 4.760 | 1.212 | 1.001 | 0.892 | 0.880 | 0.960 |
| WA | 16.001 | 1.251 | 1.238 | 1.127 | 1.446 | 1.318 |
| WI | 0.528 | 0.683 | 1.069 | 0.747 | 1.063 | 1.084 |
| WV | 1.384 | 0.786 | 0.545 | 0.626 | 0.586 | 0.636 |
| WY | 2.900 | 1.147 | 2.813 | 1.081 | 0.679 | 0.594 |

**Table S5.** AIR by state from March to August in 2020.

| **State** | **March** | **April** | **May** | **June** | **July** | **August** |
| --- | --- | --- | --- | --- | --- | --- |
| AK | 0.010 | 0.116 | 0.150 | 0.101 | 0.268 | 0.273 |
| AL | 0.022 | 0.194 | 0.954 | 0.469 | 0.861 | 0.584 |
| AR | 0.005 | 0.096 | 0.087 | 0.374 | 0.757 | 0.545 |
| AZ | 0.007 | 0.046 | 0.043 | 0.383 | 0.926 | 0.458 |
| CA | 0.001 | 0.066 | 0.144 | 0.174 | 0.495 | 0.496 |
| CO | 0.236 | 0.334 | 0.122 | 0.289 | 0.432 | 0.269 |
| CT | 0.028 | 0.243 | 0.419 | 0.183 | 0.399 | 0.379 |
| DE | 0.018 | 0.064 | 0.075 | 0.191 | 0.514 | 0.407 |
| FL | 0.009 | 0.070 | 0.127 | 0.383 | 1.134 | 0.974 |
| GA | 0.019 | 0.184 | 0.380 | 0.142 | 0.480 | 0.452 |
| HI | 0.004 | 0.091 | 0.150 | 0.105 | 0.288 | 0.359 |
| IA | 0.078 | 0.302 | 0.335 | 0.870 | 0.863 | 0.508 |
| ID | 0.198 | 0.327 | 0.366 | 0.246 | 0.500 | 0.522 |
| IL | 0.007 | 0.295 | 1.512 | 0.115 | 0.252 | 0.427 |
| IN | 0.046 | 0.439 | 0.450 | 0.232 | 0.273 | 0.376 |
| KS | 0.023 | 0.207 | 0.134 | 0.209 | 0.447 | 0.477 |
| KY | 0.018 | 0.085 | 0.031 | 0.305 | 0.668 | 0.485 |
| LA | 0.120 | 0.527 | 0.239 | 0.328 | 0.663 | 0.373 |
| MA | 0.042 | 0.394 | 0.352 | 0.183 | 0.418 | 0.354 |
| MD | 0.032 | 0.040 | 0.018 | 0.108 | 0.447 | 0.370 |
| ME | 0.032 | 0.139 | 0.159 | 0.194 | 0.350 | 0.346 |
| MI | 0.031 | 0.121 | 0.386 | 0.166 | 0.382 | 0.353 |
| MN | 0.032 | 0.220 | 0.927 | 0.453 | 0.758 | 0.450 |
| MO | 0.008 | 0.109 | 0.561 | 0.230 | 0.553 | 0.485 |
| MS | 0.014 | 0.247 | 0.641 | 0.349 | 0.791 | 0.548 |
| MT | 0.006 | 0.095 | 0.152 | 0.182 | 0.750 | 0.670 |
| NC | 0.042 | 0.110 | 0.126 | 0.257 | 0.621 | 0.487 |
| ND | 0.007 | 0.120 | 0.169 | 0.173 | 0.760 | 0.735 |
| NE | 0.017 | 1.179 | 1.702 | 0.273 | 0.367 | 0.377 |
| NH | 0.034 | 0.137 | 0.359 | 0.190 | 0.417 | 0.369 |
| NJ | 0.086 | 0.174 | 0.097 | 0.178 | 0.283 | 0.295 |
| NM | 0.006 | 0.043 | 0.039 | 0.043 | 0.305 | 0.343 |
| NV | 0.009 | 0.154 | 0.329 | 0.164 | 0.294 | 0.219 |
| NY | 0.090 | 0.463 | 0.584 | 0.214 | 0.359 | 0.302 |
| OH | 0.005 | 0.049 | 0.027 | 0.082 | 0.321 | 0.264 |
| OK | 0.013 | 0.141 | 0.817 | 0.181 | 0.442 | 0.474 |
| OR | 0.046 | 0.140 | 0.191 | 0.532 | 0.885 | 0.600 |
| PA | 0.030 | 0.165 | 0.191 | 0.183 | 0.379 | 0.316 |
| RI | 0.012 | 0.099 | 0.091 | 0.192 | 0.465 | 0.403 |
| SC | 0.101 | 0.271 | 0.195 | 0.286 | 0.545 | 0.350 |
| SD | 0.005 | 0.069 | 0.082 | 0.210 | 0.344 | 0.401 |
| TN | 0.015 | 0.382 | 0.340 | 0.299 | 0.506 | 0.491 |
| TX | 0.009 | 0.024 | 0.004 | 0.056 | 0.246 | 0.215 |
| UT | 0.090 | 0.237 | 0.165 | 0.466 | 0.572 | 0.340 |
| VA | 0.014 | 0.114 | 0.058 | 0.186 | 0.453 | 0.412 |
| VT | 0.088 | 0.158 | 0.157 | 0.173 | 0.403 | 0.379 |
| WA | 0.295 | 0.163 | 0.194 | 0.218 | 0.661 | 0.520 |
| WI | 0.010 | 0.089 | 0.168 | 0.145 | 0.486 | 0.428 |
| WV | 0.026 | 0.102 | 0.086 | 0.121 | 0.268 | 0.251 |
| WY | 0.054 | 0.149 | 0.442 | 0.209 | 0.310 | 0.234 |
